# Urolithin A targets the AKT/WNK1 axis to induce autophagy and exert anti-tumor effects in cholangiocarcinoma

**DOI:** 10.3389/fonc.2022.963314

**Published:** 2022-09-23

**Authors:** Hidenori Sahashi, Akihisa Kato, Michihiro Yoshida, Kazuki Hayashi, Itaru Naitoh, Yasuki Hori, Makoto Natsume, Naruomi Jinno, Kenta Kachi, Go Asano, Tadashi Toyohara, Yusuke Kito, Sudhakar Ammanamanchi, Hiromi Kataoka

**Affiliations:** ^1^ Department of Gastroenterology and Metabolism, Graduate School of Medical Sciences, Nagoya City University, Nagoya, Japan; ^2^ Department of Internal Medicine, University of Arizona College of Medicine, Phoenix, AZ, United States

**Keywords:** Urolithin A, UA, cholangiocarcinoma, autophagy, WNK1

## Abstract

Urolithin A (UA; 3,8-dihydroxybenzo[c]chromen-6-one), a metabolite generated by intestinal bacteria during the biotransformation of ellagitannins, has gained considerable attention in treating several cancers. Cholangiocarcinoma (CCA) remains one of the most lethal cancers; it grows in a special environment constantly exposed to both blood and bile. Since UA is known to undergo enterohepatic recirculation, we hypothesized that UA might have significant antitumor effects in CCA. Here, we investigated the therapeutic potential of UA in CCA and aimed to elucidate its mechanisms, including autophagy. UA treatment inhibited cell proliferation and induced G2/M phase cell cycle arrest in CCA cells. UA also suppressed cell migration and invasion, but did not cause apoptosis. Furthermore, Western blotting and immunocytochemistry demonstrated increased LC3-II accumulation, while electron microscopy demonstrated induced autophagosomes after UA treatment, suggesting that UA upregulated autophagy in CCA cells. In xenograft mice treated with UA, tumor growth was inhibited with increased LC3-II levels. On the other hand, phospho-kinase array demonstrated downregulation of the AKT/WNK1 pathway. LC3-II expression was elevated in WNK1 knocked down cells, indicating that WNK1 is the key signal for regulating autophagy. Thus, UA exerted antitumor effects by suppressing the AKT/WNK1 signaling pathway and inducing autophagy. In conclusion, UA, a natural, well-tolerated compound, may be a promising therapeutic candidate for advanced CCA.

## Introduction

Natural compounds have been extensively researched over the past several decades for their potential in cancer prevention and treatment (1). Ellagitannins (ETs) are naturally occurring polyphenolic compounds with a wide range of pharmacological effects, including antioxidant, anti-inflammatory, and antitumor effects (2, 3). ETs are hydrolyzed in the gut to release ellagic acid (EA), mainly present in pomegranates, strawberries, blueberries, nuts, and dried fruits (4). However, the absorption of EA is limited due to its hydrophobic nature (5).

Urolithins are metabolites of EA produced by the intestinal bacteria (6). Urolithins are much better absorbed than ETs and EA, and may provide various health benefits such as anti-obesity, antimicrobial, anti-inflammatory, anti-tumor effects (7–9). Various types of urolithins have been identified, including urolithin A (UA; 3,8-dihydroxybenzo[c]chromen-6-one), B (UB), C, and D (10, 11). Urolithins are produced in the gut from tetrahydroxy-urolithin by removal of one of the lactone rings of ellagic acid, and the subsequent removal of a hydroxyl group, resulting in the formation of UA and UB (11). Of these, UA is the major microbial metabolite observed in human, which possess anti-inflammatory and antioxidant properties (12, 13). UA has been found to induce mitophagy efficiently and improve mitochondrial function in the model organism, Caenorhabditis elegans (14). In addition, antitumor effects of UA on lung, prostate, colon, bladder, pancreatic, and neuroblastoma cancers have also been demonstrated (15–21). Several reports indicate that UA induces autophagy, but not mitophagy, *in vitro* and *in vivo* (18, 22, 23). Espín et al. reported the pharmacokinetics and tissue distribution of urolithins in Iberian pigs, which feed on oak acorns rich in ellagitannins (24). An analysis of urolithins in plasma, urine, bile, jejunum, colon, and feces revealed that UA undergoes enterohepatic recirculation and, therefore, persists in the body for long periods (24).

Cholangiocarcinoma (CCA) is the second most common primary hepatic malignancy, accounting for 10–20% of newly-diagnosed liver cancers with features of biliary tract differentiation (25, 26). Unfortunately, most CCAs are diagnosed at an advanced stage and have to be treated with systemic chemotherapy instead of surgery. However, effective chemotherapy for CCA is still limited, and the development of new therapies is required. Since CCA grows in a special environment that is constantly exposed to both blood and bile, we hypothesized that UA would have significant antitumor effects in CCA because of enterohepatic recirculation. Despite promising effects in other cancers, the antitumor effects of UA in CCA are currently unknown. We aimed to investigate the antitumor effects of UA in CCA and elucidate its mechanism, including autophagy.

## Materials and methods

### Cell cultures

Human intrahepatic cholangiocarcinoma cell lines, HuCCT-1 and SSP-25, were obtained from the RIKEN cell bank. All cell lines were cultured in RPMI-1640 medium (FUJIFILM Wako Pure Chemical Corp., Osaka, Japan), supplemented with 10% fetal bovine serum (FBS), in an incubator with 5% CO2 at 37°C.

### Cell viability assays

Cell viability was measured using a Cell Counting Kit-8 assay (Dojindo, Kumamoto, Japan), and evaluated by the absorption of WST‐1. The cells were seeded at a density of 4.0 × 10^3^ cells/well on 96-well plates. After overnight incubation, the cells were treated with or without different concentrations of UA (Cayman Chemical Co., Ann Arbor, MI, USA) for 48 h.

### Wound-healing assay (scratch assay)

The cells were grown to confluence in 12-well plates, and then a straight wound was made using a sterile 200-μL pipette tip. UA (10 or 40 μmol/L) was then added to the cells. The straight wound was photographed and measured under a microscope at 0 and 12 h. These investigations were independently performed three times.

### Transwell invasion assay

Transwell assay was performed using Corning^®^Matrigel^™^ Invasion Chamber with 8.0-μm pore membranes (top chamber) for the 24-well culture plate (Corning, NY, USA). The cells were seeded at a density of 2 × 10^5^ (HuCCT-1) cells or 1 × 10^5^ (SSP-25) cells with serum-free FBS in the top chamber of the 24-well plate, and treated with or without UA (10 or 40 μmol/L). Complete medium was added to the lower chamber. After incubation for 24 h, the invading cells were fixed with 10% formalin, stained with crystal violet, and microscopically counted.

### Western blot analysis

The cells were lysed in lysis buffer, and 20 µL of protein lysate sample was fractionated on polyacrylamide gels (TGX™ FastCast™ Acrylamide Kit; Bio-Rad Laboratories, Hercules, CA, USA) and then electroblotted to nitrocellulose membranes. The membranes were blocked with 5% skim milk in phosphate buffered saline-Tween 20 (PBS-T). The membranes were incubated with primary and then secondary antibodies. They were then treated with enhanced chemiluminescence detection reagents (Amersham™; Cytiva, Marlborough, MA, USA), and chemiluminescent signals were visualized as bands using a LAS 4000 mini analyzer (Cytiva).

Antibodies against phospho-cdc2 (Try15), cyclin D1, cyclin B1, cleaved caspase-3, caspase-3, phospho-AKT (Ser473), AKT, phospho-WNK1 (Thr60), WNK1, phospho-GSK-3β (Ser9), GSK-3β, phospho-mTOR (Ser2448), and mTOR were purchased from Cell Signaling Technology (Beverly, MA, USA). Monoclonal beta-actin antibody (FUJIFILM Wako Pure Chemical Corp.) was used to probe an internal control.

### Flow cytometry analysis

The cells were seeded in 60-mm dishes and cultured overnight, and then treated with or without UA (40 μmol/L) for 24, 48 and 72 h. After treatment, floating and attached cells were collected and stained, and flow cytometric analysis was performed using a flow cytometer (FACSCanto II, BD Biosciences; San Jose, CA, USA). Cell cycles were evaluated by PI staining (PI solution, Dojindo) and apoptosis was detected using the Annexin V Cell Apoptosis Detection Kit 1 (BD Biosciences) according to the manufacturer’s instructions. Camptothecin (Merck, Darmstadt, Germany) was used as a positive control for the apoptosis assay.

### Detection of autophagy

Autophagic cells were detected with LC3 using autophagy watch (Medical & Biological Laboratories, Aichi, Japan), according to the manufacturer’s instructions. For Western blot analysis, HuCCT-1 and SSP-25 cells were treated with UA (40 μmol/L) and/or Chloroquine (CQ; 20 μmol/L) for 24 h, and the analysis was performed with 20 µL of protein lysate sample using anti-LC3 monoclonal antibody-HRP-DirecT (Autophagy watch). For immunocytochemistry, the cells were evaluated using anti-LC3 monoclonal antibody (Autophagy watch), with Alexa Fluor 488-conjugated goat anti-rabbit IgG (H + L; Thermo Fisher Scientific, Waltham, MA, USA) as the secondary antibody. All sections were counterstained using 4′,6-diamidino-2-phenylindole (DAPI; Fluoromount-G; Southern Biotech, Birmingham, AL, USA). HuCCT-1 cells were seeded in 4-well glass slides (Lab-Tek^®^ Chamber Slide™ system; Thermo Fisher Scientific) and incubated for 24 h, and then treated under the respective conditions for 24 h. Images were obtained using a confocal laser scanning fluorescence microscope (FV3000; Olympus, Tokyo, Japan).

### Transmission electron microscopy

HuCCT-1 cells were seeded at a density of 1.5 × 10^5^ cells/well on 6-well plates. After overnight incubation, the cells were treated with UA (40 μmol/L) and/or CQ (20 μmol/L) for 24 h, and the samples were pre-fixed with 2% glutaraldehyde in 0.1 M phosphate buffer (pH 7.4) at 4°C. After fixation, the specimens were post-fixed with 2% osmium tetroxide in 0.1 M phosphate buffer (pH 7.4) for 45 min. They were subsequently dehydrated in a graded series of ethanol and embedded in epoxy resin. Ultra-thin sections were cut using an Ultracut-UCT (LEICA, Wetzlar, Germany) with a diamond knife, and stained with 2% uranyl acetate in distilled water for 15 min followed by a lead staining solution for 5 min. Sections were examined with a JEM-1400 plus (JEOL, Tokyo, Japan) electron microscope.

### Human Phospho-kinase array

Phosphorylated proteins were analyzed using the Human Phospho-Kinase Array Kit (ARY003C; R&D Systems, Minneapolis, MN, USA). HuCCT-1 and SSP-25 cells were treated with or without UA (40 μmol/L) for 3 h and according to the manufacturer’s instructions. Signals were detected using chemiluminescence detection reagents (Amersham™, Cytiva), and array images were analyzed using the ImageJ software.

### Transfection

Small interfering RNA (siRNA) transfection was performed using Lipofectamine RNAi-MAX (Thermo Fisher Scientific) according to the manufacturer’s instructions. HuCCT-1 cells were transfected with the desired siRNA using siGENOME non-targeting siRNA (siNT) control pool and siGENOME human WNK1 siRNA SMART pool (Dharmacon, Lafayette, CO, USA). Two days after transfection, the cells were treated with each condition for 3 or 24 h.

### *In vivo* experiments

The protocols for all animal studies were approved by Nagoya City University Center for Experimental Animal Science, and the mice were housed according to the guidelines of Nagoya City University for Animal Experiments. Female nude mice (BALB/c Slc-nu/nu), aged 7 weeks, were obtained from Japan SLC Inc. The mice were acclimatized for 2 weeks before the experiments, and were kept in individual cages with unrestricted access to food and water. All mice were maintained under specific pathogen-free conditions with a 12-h light/dark cycle. To prepare the xenograft models, HuCCT-1 cells were injected into the mouse flanks with 5 × 10^6^ cells in 100 μL of media. One day after implantation, the mice were randomly allocated into two groups. Two weeks after subcutaneous tumor transplantation, UA (20 mg/kg, 3 times a week) or dimethyl sulfoxide (DMSO; control) was administered by oral gavage, as in a previous study (20). The maximum tumor diameter (L) and the diameter at right angles to that axis (W) were measured using calipers twice a week, and the tumor volume was calculated according to the formula: (L×W2)/2. The weights of the mice were also recorded twice a week. The mice were sacrificed 35 days after the start of medication, and the transplanted tumors were excised and fixed in formalin or frozen in liquid nitrogen for protein lysate.

### *In vivo* immunohistochemistry

The tumors were excised, and immediately fixed in formalin and embedded in paraffin blocks. Then, the block specimens were sectioned (4 µm) and stained using Ki-67 antibodies (Cell Signaling Technology). High spot areas were captured under a microscope, and the positive areas were counted visually. Data were expressed as means ± SD (Standard Deviation) of five independent experiments.

### Statistical analysis

The data were analyzed using Student’s *t* test and Mann-Whitney *U* test. Differences were considered statistically significant at *P* <.05. Data were expressed as means ± SD.

## Results

### UA treatment inhibited cell proliferation and induced G2/M phase cell cycle arrest in cholangiocarcinoma cell lines

**Figure 1:**
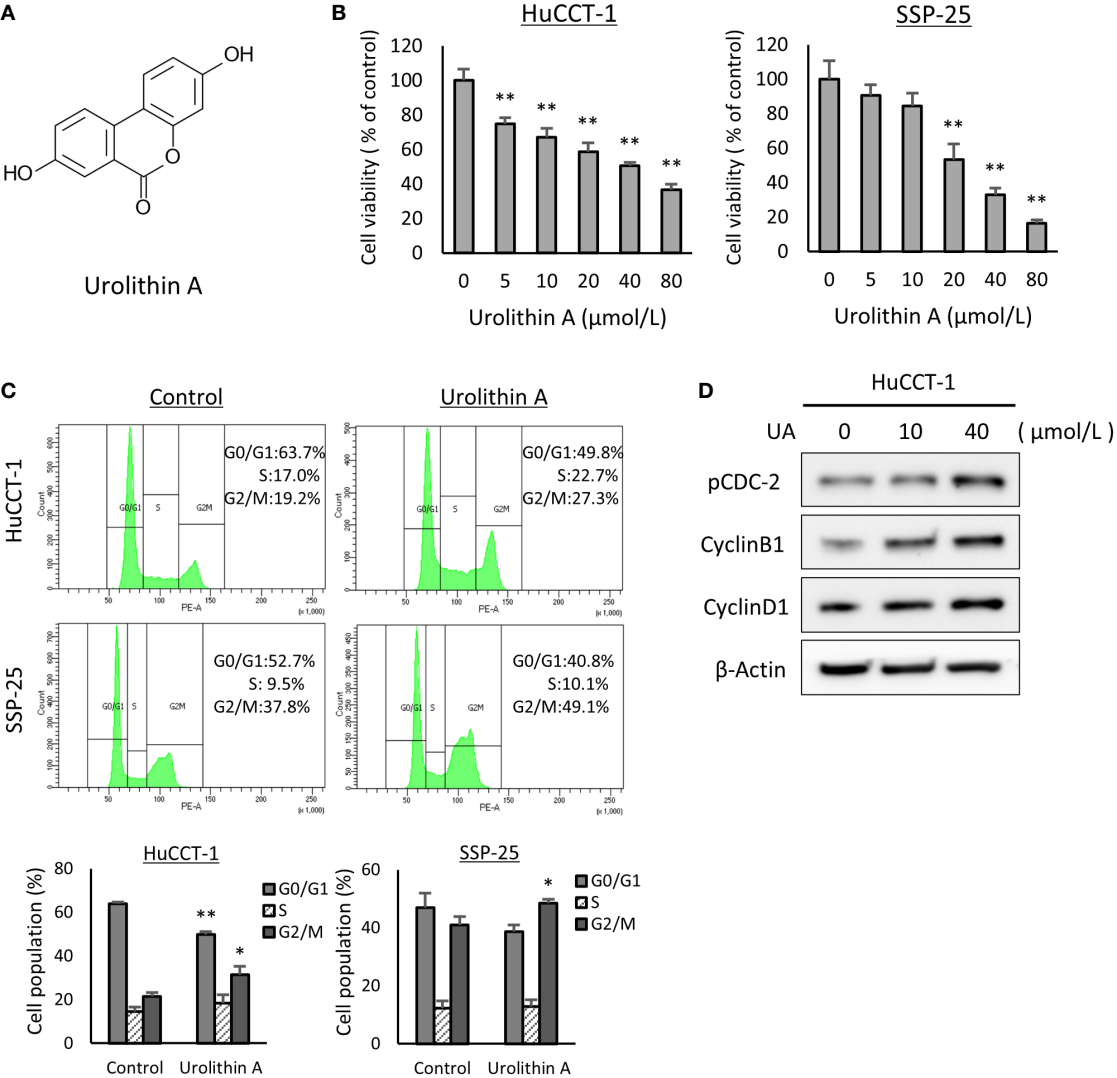
UA treatment inhibits cell proliferation and induces G2/M phase cell cycle arrest in cholangiocarcinoma cell lines. **(A)** Chemical structures of UA. **(B)** HuCCT-1 and SSP-25 cells were treated with UA at 0–80 μmol/L for 48 h Cell viability was measured using the Cell Counting Kit-8 assay. Data represent the means of three independent experiments. Bars, standard deviation; **P < 0.01. **(C)** HuCCT-1 and SSP-25 cells were treated with 0 or 40 μmol/L UA for 48 h Cell cycles were determined using flow cytometry. Data represent the means of three independent experiments. Bars, standard deviation; *P < 0.05; **P < 0.01. **(D)** HuCCT-1 were treated with 0, 10, or 40 μmol/L UA for 48 h Expression of cell cycle regulators was analyzed by Western blotting for phospho (p)-cdc2 (Try15), cyclin B1, and Cyclin D1. β-actin was used as internal loading control.

The chemical structure of UA is shown in **Figure 1A**. To assess sensitivity for UA, a cell viability assay was performed with HuCCT-1 and SSP-25 cells. We found that the viabilities of the two cell lines treated with UA for 48 h were reduced in a dose-dependent manner (**Figure 1B**). We further explored the effect of UA on the cell cycle using flow cytometry (FACS). HuCCT-1 and SSP-25 cells treated with 40 μmol/L UA for 48 h showed accumulation of cells in the G2/M phase compared to the controls (control vs. 40 μmol/L UA in HuCCT-1 cells: 21.3± 1.9% vs. 31.5 ± 3.7%; and in SSP-25 cells: 40.9 ± 3.9% vs. 48.5 ± 1.3%, respectively, P < 0.05) (**Figure 1C**). As shown in **Supplementary Figure 1**, the G2/M phase cell accumulation was also observed under the conditions treated with UA for 24 or 72 h. Then, to confirm major cell cycle regulators of the G2/M phase, we examined the changes in phospho-cdc2 (Try15), cyclin B1, and cyclin D1 using Western blot analysis. HuCCT-1 cells treated with UA for 48 h upregulated the expression of phospho-CDC2 (Tyr15) and cyclin B1 without influencing cyclin D1 levels, consistent with the observed G2/M cell cycle arrest (**Figure 1D**).

### Effects of UA on cell migration, invasion, and apoptosis progression in cholangiocarcinoma cell lines

**Figure 2:**
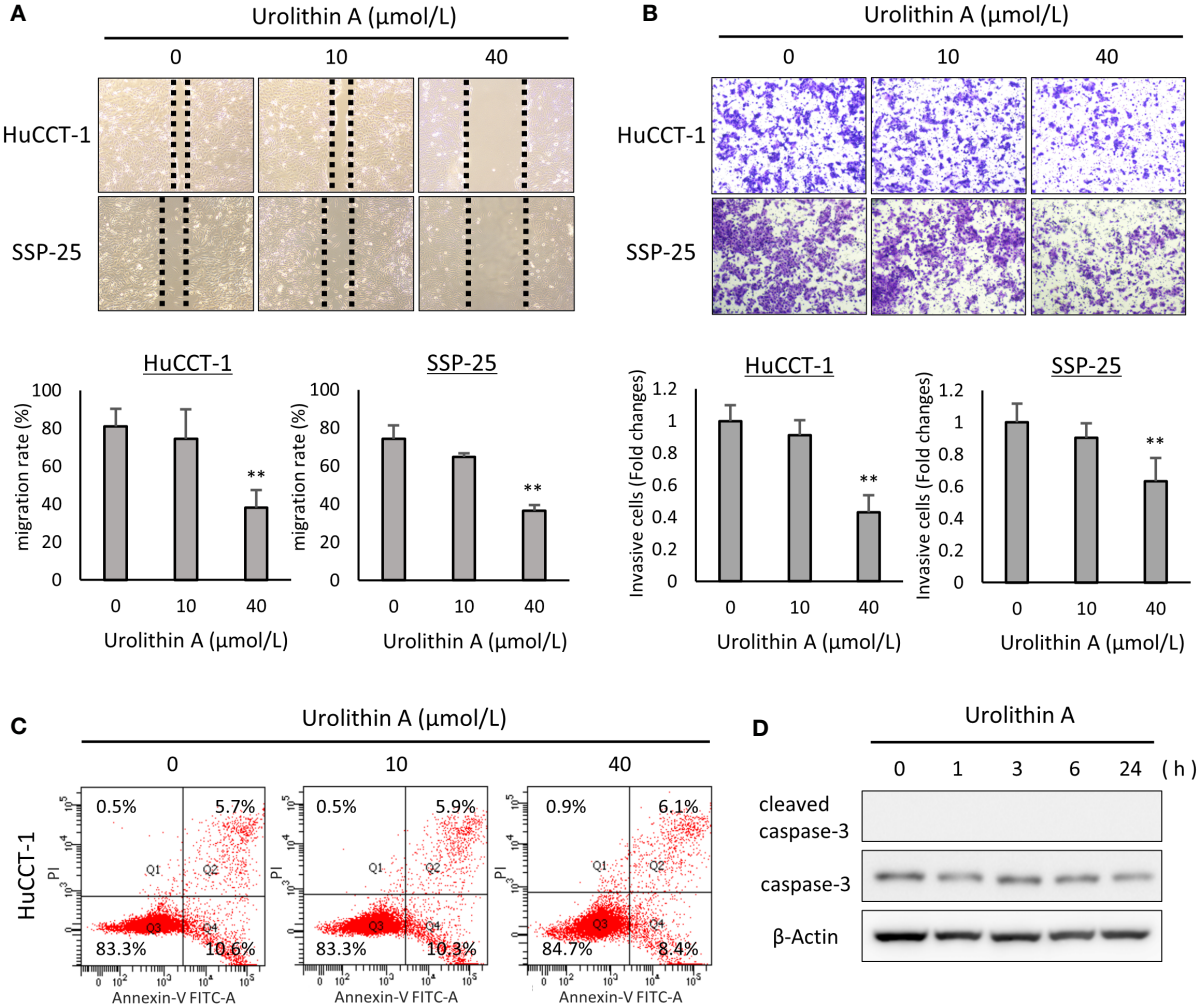
Effects of UA on cell migration, invasion, and apoptosis progression in cholangiocarcinoma cell lines. **(A)** Representative images obtained at 12 h after a scratch wound was made in confluent monolayers of HuCCT-1 and SSP-25 cells. After the scratch, 0, 10, or 40 μmol/L of UA were added. The migration rates were quantified by measuring the area of the injured region. Data represent the means of three independent experiments. Bars, standard deviation; **P < 0.01. **(B)** Representative transwell-membrane images stained with crystal violet show invasion cells after 12 h of treatment with 0, 10, or 40 μmol/L UA in HuCCT-1 and SSP-25 cells. Quantitative analysis of the invasion cells was expressed as fold change relative to untreated controls. Data represent the means of three independent experiments. Bars, standard deviation; **P < 0.01. **(C)** HuCCT-1 cells were treated with 0, 10, or 40 μmol/L UA for 24 h, and then stained with annexin-V FITC and PI. Apoptosis cells were evaluated using flow cytometry. **(D)** HuCCT-1 cells were treated with 40 μmol/L UA for 0, 1, 3, 6, or 24 h Expression of apoptosis-related factors was analyzed by Western blotting for cleaved caspase-3 and caspase-3. β-actin was used as an internal loading control.

To evaluate the effects of UA on cell migration, we conducted a wound-healing assay. UA treatment (40 μmol/L) significantly suppressed cell migration in both HuCCT-1 (0, 10, and 40 μmol/L UA: 81.2 ± 9.0%, 74.6 ± 15.5%, and 38.1 ± 9.3%, respectively, P < 0.01) and SSP-25 (0, 10, and 40 μmol/L UA: 74.1 ± 7.1%, 64.8 ± 1.9%, and 36.6 ± 3.0%, respectively, P < 0.01) cells (**Figure 2A**). We also performed the transwell assay to evaluate the effects of UA on cell invasion. UA significantly inhibited cell invasion at 40 μmol/L in both HuCCT-1 (0, 10 and 40 μmol/L UA: 1.0 ± 0.097, 0.91 ± 0.094, and 0.43 ± 0.106, respectively, P < 0.01) and SSP-25 (0, 10, and 40 μmol/L UA: 1.0 ± 0.119, 0.90 ± 0.091, and 0.63 ± 0.143, respectively, P < 0.01) cells (**Figure 2B**).

To investigate the effects of UA on apoptosis, we used the AnnexinV-FITC/PI staining method with flow cytometry. As shown in **Supplementary Figure 2**, 30 μmol/L Camptothecin for 24 h was used as a positive control for the apoptosis assay. Interestingly, there was no difference between control and UA treatment in the percentage of apoptotic cells in HuCCT-1 cells, treated with or without UA for 24h (0, 10, and 40 μmol/L UA: 10.6%, 10.3% and 8.4%) (**Figure 2C**). And, as shown **in Supplementary Figure 3**, there was also no difference between them under the conditions treated with UA for 48 or 72 h. We also examined the effects of UA on apoptosis-related factors, total and cleaved caspase-3, using Western blot analysis. There were no apparent changes in the total and cleaved caspase-3 in HuCCT-1 cells treated with 40 μmol/L UA for 0, 1, 3, 6, or 24 h (**Figure 2D**).

### UA-mediated upregulation of autophagy in cholangiocarcinoma cells

**Figure 3:**
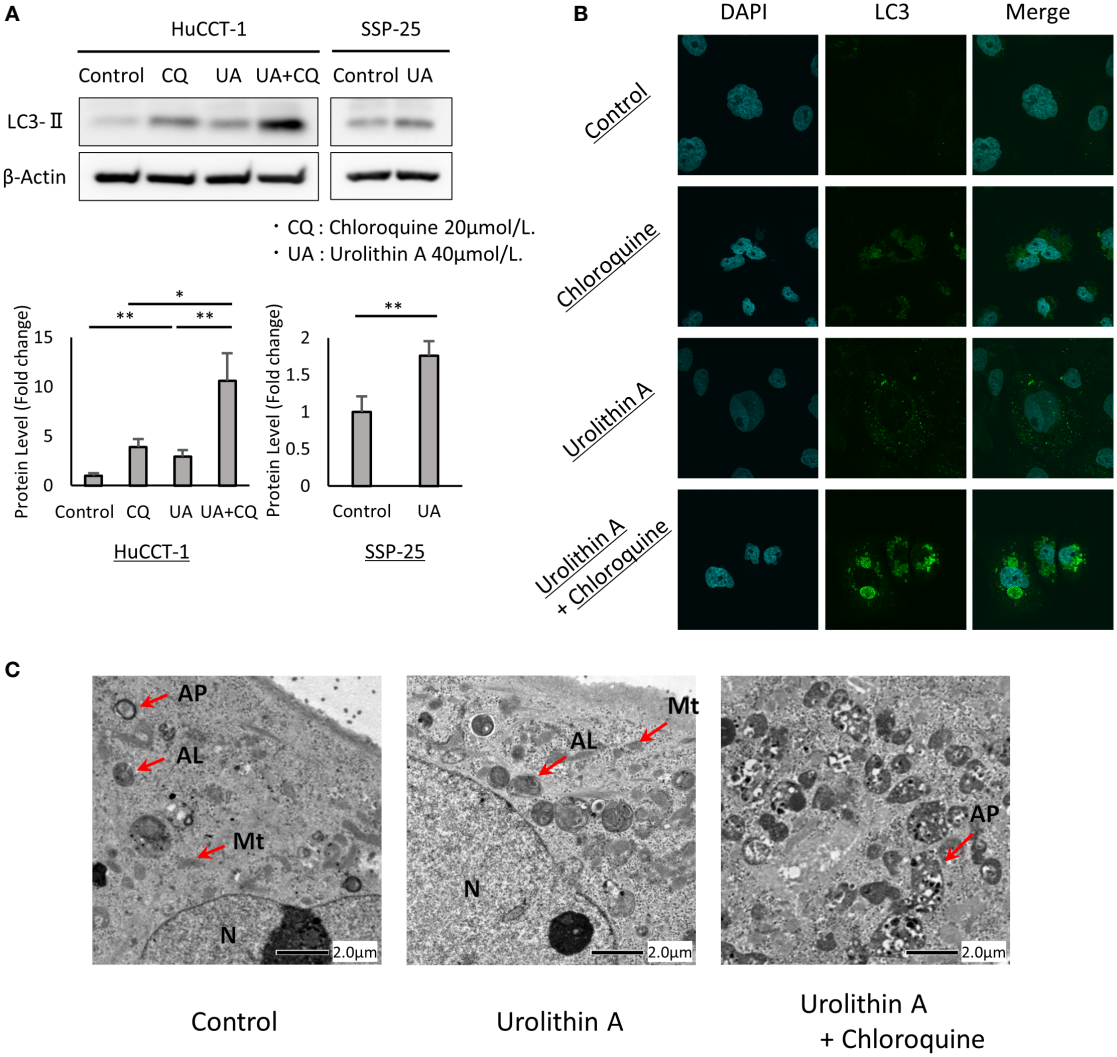
UA-mediated upregulation of autophagy in cholangiocarcinoma cells. **(A)** HuCCT-1 and SSP-25 cells were treated with 20 μmol/L CQ and 40 μmol/L UA for 24 h Autophagy was detected by Western blotting for LC3-II. β-actin was used as an internal loading control. LC3-II levels were normalized against β-Actin and represented the means of three independent experiments. Bars, standard deviation; *P < 0.05; **P < 0.01. **(B)** Immunofluorescence for LC3 (green) was performed after the same treatment as shown in **(A)**. Blue staining denotes DAPI-labeled nuclei. **(C)** Electron microscopy after the same treatment as shown in **(A)**. N, nucleus; Mt, mitochondrion; AP, autophagosome; AL, autolysosome.

Increased LC3-II levels and the formation of LC3 puncta were used to determine whether UA treatment induced autophagy in cholangiocarcinoma cells. To confirm the contribution of UA treatment to autophagy, we performed autophagy flux assay with CQ, which blocks the fusion of autophagosomes with lysosomes and inhibits late-stage autophagy. We first examined the effects of UA on autophagy using Western blot analysis. It was found that UA treatment for 24 h caused an increase in LC3-II levels in HuCCT-1 and SSP-25 cells. CQ induced LC3-II expression, and addition of UA led to further accumulation of LC3-II in HuCCT-1 cells (**Figure 3A**). In addition, immunofluorescent staining revealed that UA, CQ, and their combination significantly increased LC3 puncta accumulation in the cytoplasm of cells compared to control (**Figure 3B**). Furthermore, transmission electron microscopy (TEM) demonstrated that there were more autophagosomes and autolysosomes in HuCCT-1 cells treated with UA for 24 h. After combined treatment with UA and CQ, autophagosomes that had stopped prematurely were clearly observed in the cytoplasm (**Figure 3C**).

### UA inhibited xenograft tumor growth *in vivo*

**Figure 4:**
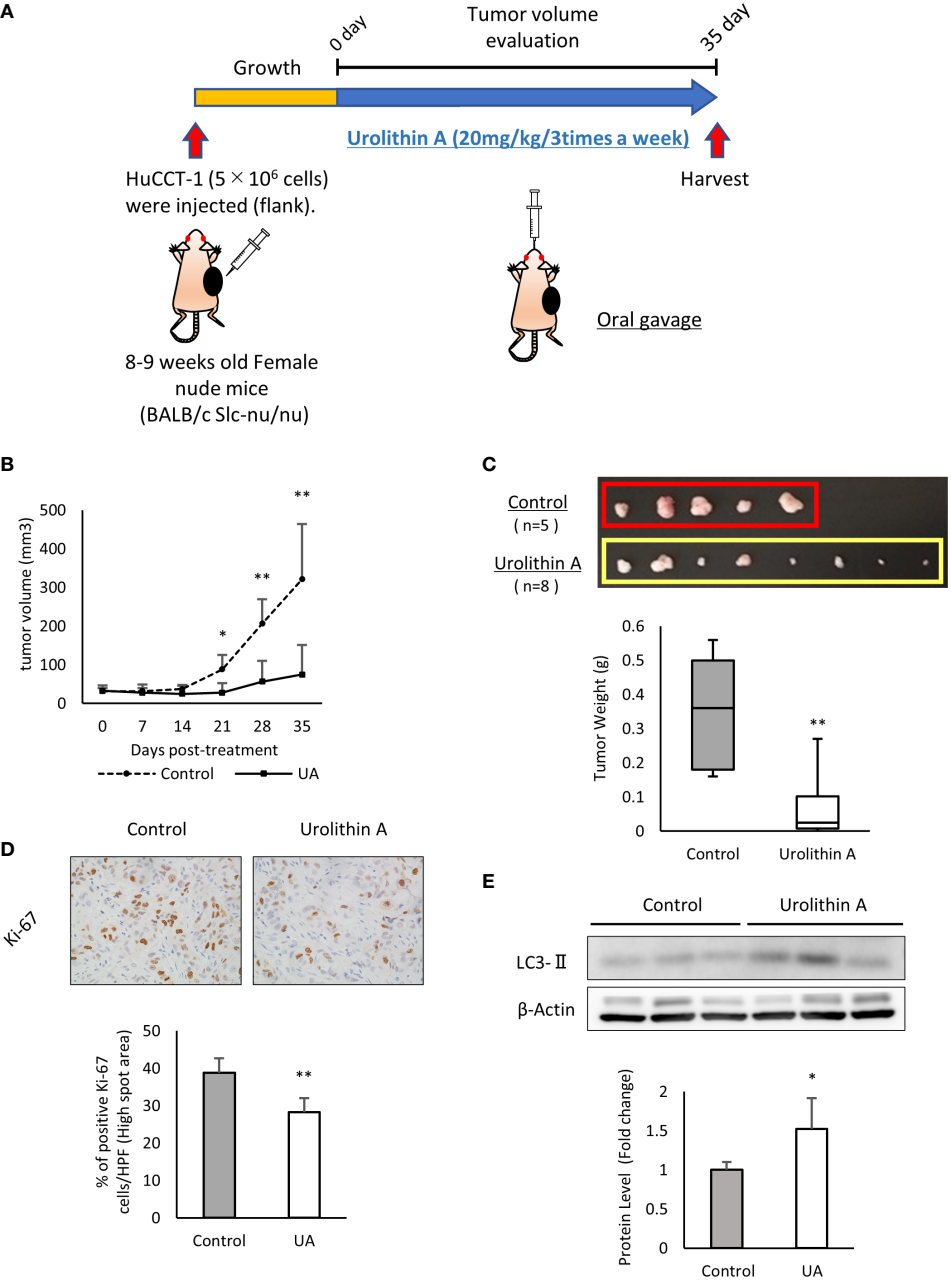
UA inhibits xenograft tumor growth *in vivo*. **(A)** Experimental design for UA treatment in the xenograft model. HuCCT-1 cells were injected into the flank of nude mice. UA (20 mg/kg, 3 times a week) or DMSO (control) were administered by oral gavage for 35 days. **(B)** The volume of subcutaneous tumors in the xenograft model was measured twice a week. Data represent the means of the control group (n = 5) or the UA group (n = 8). Bars, standard deviation; *P < 0.05; **P < 0.01. **(C)** Representative macroscopic images of tumors in nude mice obtained at day 35 after the start of the treatment. Data represent the means of the control group (n = 5) or the UA group (n = 8). Bars, standard deviation; **P < 0.01. **(D)** Representative Ki67-stained immunohistochemical images of the two groups. The positive rates of Ki67 staining were quantified by measuring five high spot areas from each tumor. Data represent the means of the control group (n = 4) or the UA group (n = 4). Bars, standard deviation; **P < 0.01. **(E)** Autophagy was detected by Western blotting for LC3-II in the two groups. β-actin was used as an internal loading control. LC3-II levels were normalized against β-Actin and represented the means of three independent experiments. Bars, standard deviation; *P < 0.05.

The above-mentioned results demonstrated the efficacy of UA in cholangiocarcinoma cells. To verify these effects *in vivo*, we subcutaneously injected HuCCT-1 cells into the flank of nude mice as xenograft models. UA (20 mg/kg, 3 times a week) or DMSO (control) was administered by oral gavage for 35 days (**Figure 4A**). There was no body weight loss in the treatment group compared to the control group during the treatment (data not shown), which suggested that the volume of UA used was not harmful to the mice. Tumor volume and weight significantly reduced in the UA-treated mice compared to controls (**Figures 4B, C**). The proliferative potential of mice tumor samples were analyzed by Ki-67 immunostaining. The number of Ki-67 positive cells in the high spot area was significantly suppressed in the UA treatment group compared to the control group (**Figure 4D**). Western blot analysis revealed that the UA treatment group had significantly higher LC3-II levels than the control group (**Figure 4E**). These results suggested that UA could suppress tumor growth and might induce autophagy in cholangiocarcinoma.

### UA treatment downregulated AKT and WNK1 pathways, and induced autophagy in cholangiocarcinoma cells

**Figure 5:**
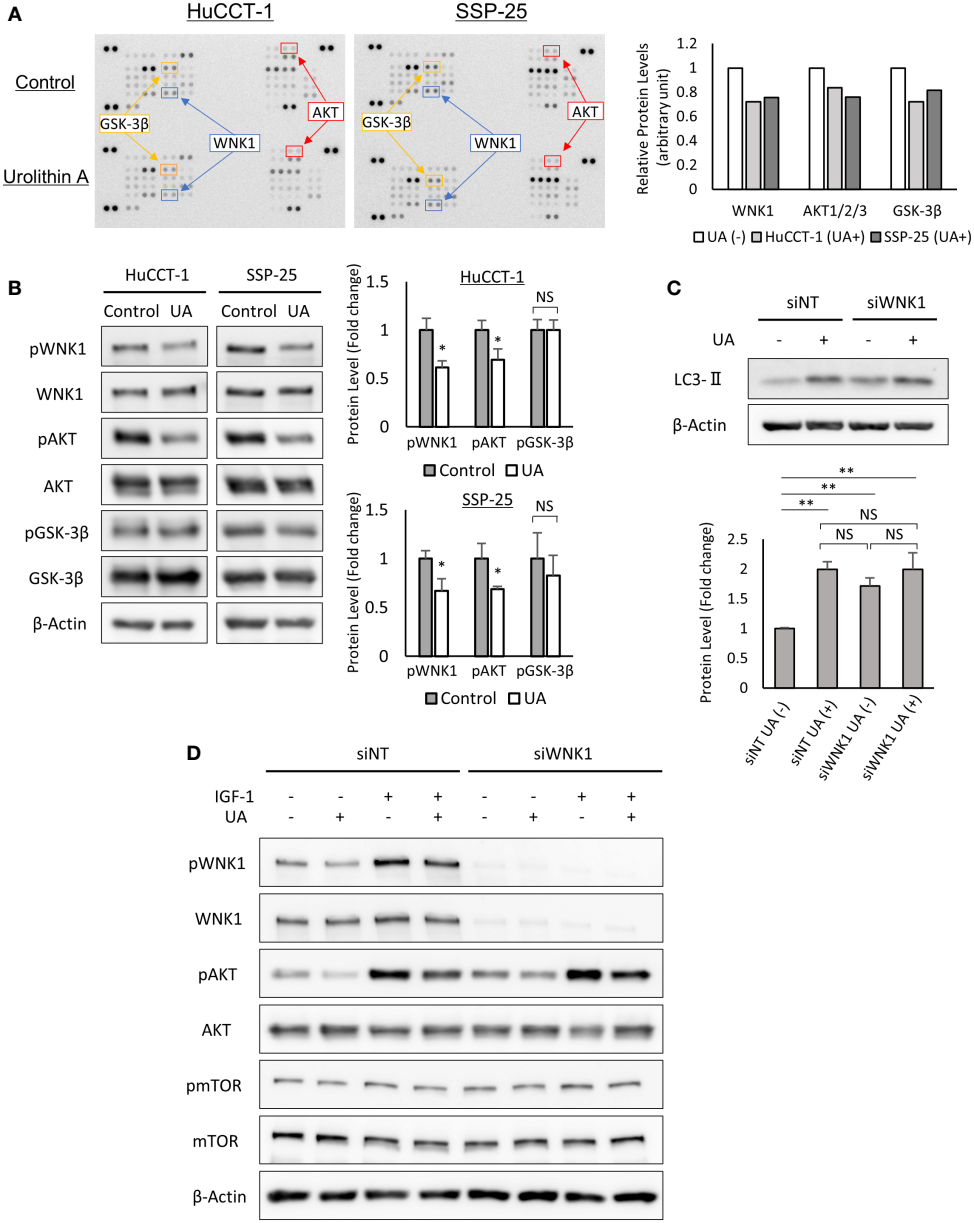
UA treatment downregulated AKT and WNK1 pathways, and induced autophagy in cholangiocarcinoma cells. **(A)** HuCCT-1 and SSP-25 cells were treated with 40 μmol/L UA for 3 h and analyzed using the human Phospho-Kinase array. Relative levels of protein phosphorylation (normalized intensity for each antibody) were quantified as a ratio of the UA-treated sample to the untreated one. **(B)** Results of the human Phospho-Kinase array were verified by Western blotting. β-actin was used as an internal loading control. Protein phosphorylation levels were normalized against β-Actin and represented the means of three independent experiments. Bars, standard deviation; NS, not significant; *P < 0.05. **(C)** Western blotting for LC3-II in WNK1 knocked down HuCCT-1 cells. Cells were treated with 40 μmol/L UA for 24 h β-actin was used as an internal loading control. LC3-II levels were normalized against β-Actin and represented the means of three independent experiments. Bars, standard deviation; NS, not significant; **P < 0.01. **(D)** Western blotting for WNK1 (Thr60 and total), mTOR (Ser2448 and total), and AKT (Ser473 and total) in HuCCT-1 cells transfected with control (siNT) or WNK1-specific (siWNK1) small interfering RNAs. Cells were treated with 40 μmol/L UA and 50 ng/mL IGF-1 for 3 h.

To clarify the key regulatory pathways of UA treatment, we utilized the human Phospho-kinase array. UA treatment downregulated the expressions of phospho-WNK1, phospho-AKT, and phospho-GSK-3β in HuCCT-1 and SSP-25 cells (**Figure 5A**). The significant changes of phosphorylation for WNK1 and AKT were also confirmed in the two cell lines using Western blot analysis, but were not seen in GSK-3β (**Figure 5B**). Therefore, we hypothesized that UA treatment might induce autophagy *via* the AKT/WNK1 pathway. To verify our hypothesis, we analyzed LC3-II expression in WNK1 knocked down HuCCT-1 cells. Western blot analysis for LC3-II revealed that the targeted knockdown of WNK1 elevated LC3-II protein without UA treatment, suggesting the importance of WNK1 in the activation of autophagy. Furthermore, UA treatment indicated similar up-regulation of LC3-II, regardless of the knockdown of WNK1. These results suggested that UA induced autophagy mainly *via* the AKT/WNK1 pathway (**Figure 5C**). In addition, Western blotting with IGF-1, known as the AKT-WNK1 signal activator, demonstrated that UA downregulated phospho-WNK1 levels even in HuCCT-1 cells treated with IGF-1. We further assessed the mTOR activity, which is another pathway for induction of autophagy, by measuring phosphorylation of mTOR, and found that the mTOR pathway was not affected by UA treatment and WNK1 knockdown (**Figure 5D**).

## Discussion

Systemic chemotherapy with a combination of gemcitabine and is globally considered the standard first-line therapy for advanced CCA (27). However, effective chemotherapy for CCA is still limited, and the development of new therapies has not proressed sufficiently. Many targeted therapies for CCA, targeting FGFR2 fusions (28), IDH mutations (29, 30), major downstream pathways (31), and growth factor receptors (32), have been reported. However, clinical trials on therapies that appeared promising on basic research have not led to clinical breakthroughs due to various challenges (33).

UA is a metabolite generated by intestinal bacteria after ingestion of EA- and ET-rich foods and health supplements (34). UA is reported to have antitumor effects in many cancers, such as lung, prostate, colon, bladder, pancreatic, and neuroblastoma cancers (15–21). Given that UA undergoes enterohepatic recirculation (24), we speculated that UA might have significant antitumor effects in CCA, which grows in a special environment that is constantly exposed to both blood and bile. In this study, we demonstrated that UA showed antitumor effects by inhibiting cell viability, migration, and invasion in HuCCT-1 and SSP-25 cells (**Figure 1**). In addition, UA administration dramatically reduced tumor growth in a xenograft mice model (**Figure 4**).

The mechanism of the antitumor effects of UA is characterized by various factors that regulate intracellular molecule targets, ultimately influencing cancer cell survival. Our data suggested that UA showed antitumor effects mainly *via* autophagy in cholangiocarcinoma cells (**Figure 3**). Autophagy is a self-degradative process required to maintain cellular homeostasis, development, differentiation, survival, and death (35). In cancer, suppression or induction of autophagy can exert antitumor effects through promotion of cell death or survival, which are two main therapeutic targets (36). Thus, it is essential to identify key autophagy targets for new therapeutic agents. Previous studies have reported a cross-talk relationship between autophagy and apoptosis in anti-tumor therapy (37), but in our study, UA treatment did not lead to apoptosis (**Figure 2**). We suggest that UA significantly affects cancer cell survival by inducing autophagy in cholangiocarcinoma.

Autophagy is mainly mediated through the PI3K/Akt/mTOR and AMPK/mTOR signaling pathways, the molecular mechanisms by which mTOR kinase induces autophagy (36). We examined the change in mTOR phosphorylation after UA treatment and found that UA did not cause any change in mTOR phosphorylation (**Figure 5D**). Kankanamalage et al. reported that reduced WNK1 expression accelerates autophagy independently of the mTOR signaling pathway (38). In concordance with that report, we found that WNK1 knockdown induced autophagy regardless of the mTOR signaling pathway in HuCCT-1 cells (**Figures 5C, D** **)**.

**Figure 6:**
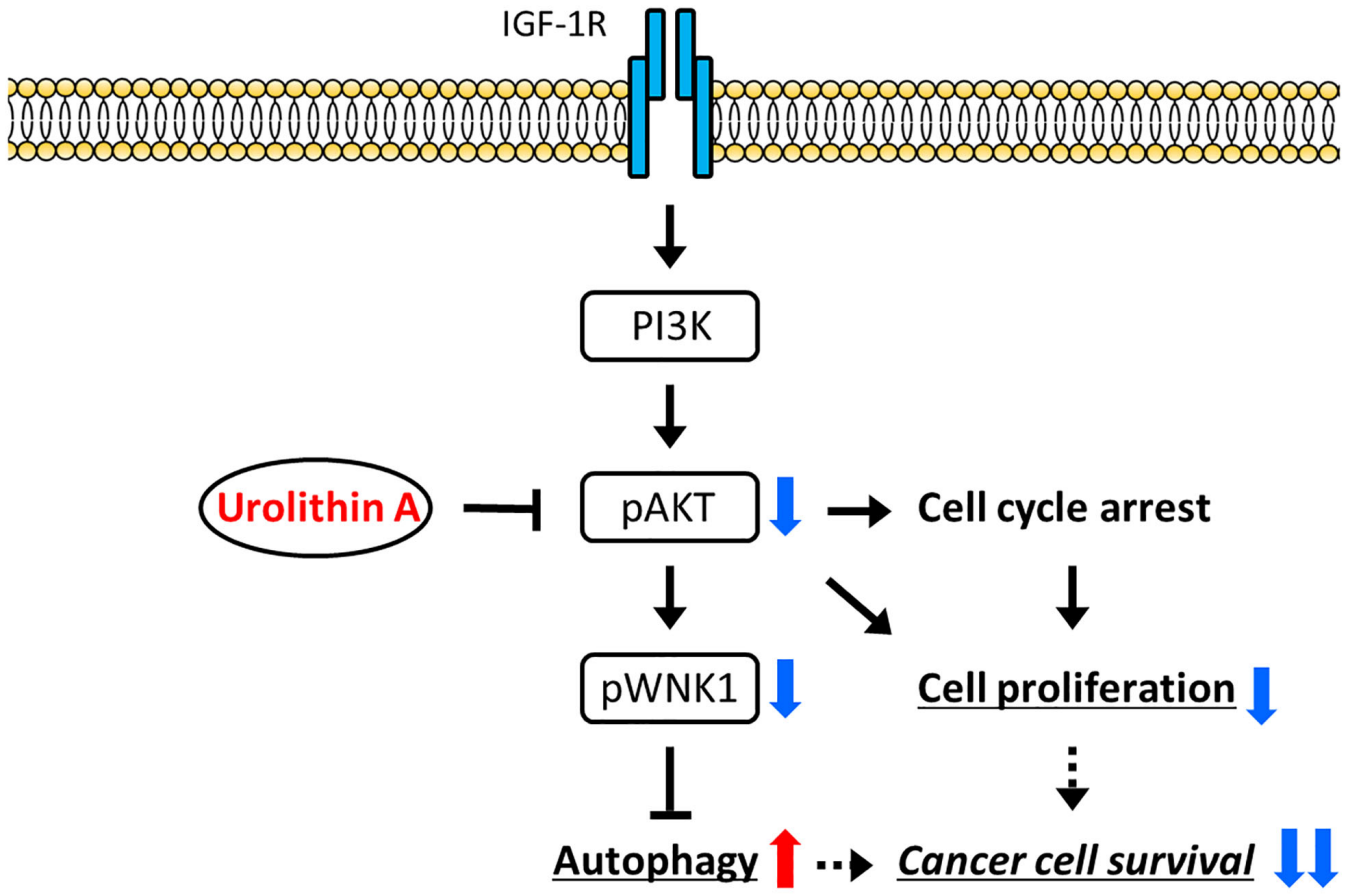
A proposed model of the mechanism. UA treatment reduces cell proliferation by inhibiting the activation of AKT, and inducing autophagy *via* the WNK1 pathway. As a result, cancer cell survival is suppressed by UA in cholangiocarcinoma cells.

WNKs (With-no-lysine kinases) are a family of four serine-threonine protein kinases, WNK1–4, with an atypical placement of the catalytic lysine (39). Initial attention was focused on these enzymes as regulators of blood pressure because mutations of two family members, WNK1 and WNK4, caused pseudohypoaldosteronism type II, a heritable form of hypertension (39). WNK1 was also reported to be involved in PI3K-AKT pathway activation in several cancers (40). Likewise, our study indicated that IGF-1 stimulation upregulated AKT phosphorylation in WNK1 knockdown cells, indicating that WNK1 was downstream of AKT (**Figure 5D**). From these results, we proposed a schematic representation of the signaling pathway involved in the inhibition of cancer growth by UA-modulation of the AKT/WNK1 axis (**Figure 6**).

According to recent pharmacokinetic studies, UA is reported to undergo phase-II metabolism, to be mainly glucuronides, after absorption (41). Several *in vitro* studies showed that UA phase-II metabolites have lower bioactivity than deconjugated UA, including anti-tumor effects and inflammation (42–44). However, in the present study, UA oral administration exerts significant anti-tumor effects in xenograft model. Some reports indicated UA glucuronides are susceptible to β-Glucuronidase, which is known to present at high concentration in the microenvironment of most solid cancers (45, 46). On the basis of these findings, we speculated that β-Glucuronidase might be related to deconjugation of UA in *in vivo* study. Further investigation is needed.

In terms of safety of UA supplementation, a human clinical study revealed that UA was biologically safe and improved mitochondrial function in older adults (47). A recent randomized, double-blind, placebo-controlled clinical study demonstrated that daily 1000-mg UA supplementation in healthy older adults for 4 months was biologically safe, and improved muscle endurance and mitochondrial health (48). In our study, the UA dose used in mice (20 mg/kg) was convertible to a human equivalent dose (HED) of approximately 1.62 mg/kg for adults (49), and is expected to be safe. The potential clinical application of UA appears promising on the basis of its safety and benefits.

Collectively, our *in vitro* and *in vivo* data revealed that UA exerted antitumor effects by suppressing the AKT/WNK1 signaling pathway and inducing autophagy. Thus, UA, a natural, well-tolerated compound, may be a promising therapeutic candidate for advanced CCA.

## Data availability statement

The original contributions presented in the study are included in the article/**Supplementary Material**. Further inquiries can be directed to the corresponding author.

## Ethics statement

The animal study was reviewed and approved by Nagoya City University Center for Experimental Animal Science.

## Author contributions

AK and HS, MY designed the study, and drafted the paper. HS mainly contributed *in vitro* and *in vivo* data on cholangiocarcinoma cells. MN and NJ, KK, GA, TT performed *in vivo* experiments. MY and IN, YH, YK analyzed the data. SA and KH, HK finalized and revised the paper. All authors contributed to the article and approved the submitted version.

## Funding

This study was supported by a JSPS KAKENHI grant number 22K15973 and Pancreas Research Foundation of Japan (to AK), and a JSPS KAKENHI grant number 20K08291 (to MY), and a JSPS KAKENHI grant numbert 17K09479 (to IN).

## Acknowledgments

We acknowledge the assistance of the Research Equipment Sharing Center at the Nagoya City University.

## Conflict of interest

The authors declare that the research was conducted in the absence of any commercial or financial relationships that could be construed as a potential conflict of interest.

## Publisher’s note

All claims expressed in this article are solely those of the authors and do not necessarily represent those of their affiliated organizations, or those of the publisher, the editors and the reviewers. Any product that may be evaluated in this article, or claim that may be made by its manufacturer, is not guaranteed or endorsed by the publisher.

## Supplementary material

Supplementary Figure 1 UA treatment inhibits cell proliferation and induces G2/M phase cell cycle arrest in cholangiocarcinoma cell lines. HuCCT-1 and SSP-25 cells were treated with 0 or 40 μmol/L UA for 24 and 72 h. Cell cycles were determined using flow cytometry. Data represent the means of three independent experiments. Bars, standard deviation; **P < 0.01.

Supplementary Figure 2 Positive control for the apoptosis assay. HuCCT-1 cells were treated with 30 μmol/L Camptothecin for 24 h, and then stained with annexin-V FITC and PI. Apoptosis cells were evaluated using flow cytometry.

Supplementary Figure 3 Effects of UA on apoptosis progression. HuCCT-1 cells were treated with 0 or 40 μmol/L UA for 48 or 72 h, and then stained with annexin-V FITC and PI. Apoptosis cells were evaluated using flow cytometry.
