# Supplementary figures and images for: Urolithin A targets the AKT/WNK1 axis to induce autophagy and exert anti-tumor effects in cholangiocarcinoma
Source: Front Oncol. 2022 Sep 23;12:963314. doi: 10.3389/fonc.2022.963314 (PMC9539031; doi:10.3389/fonc.2022.963314)

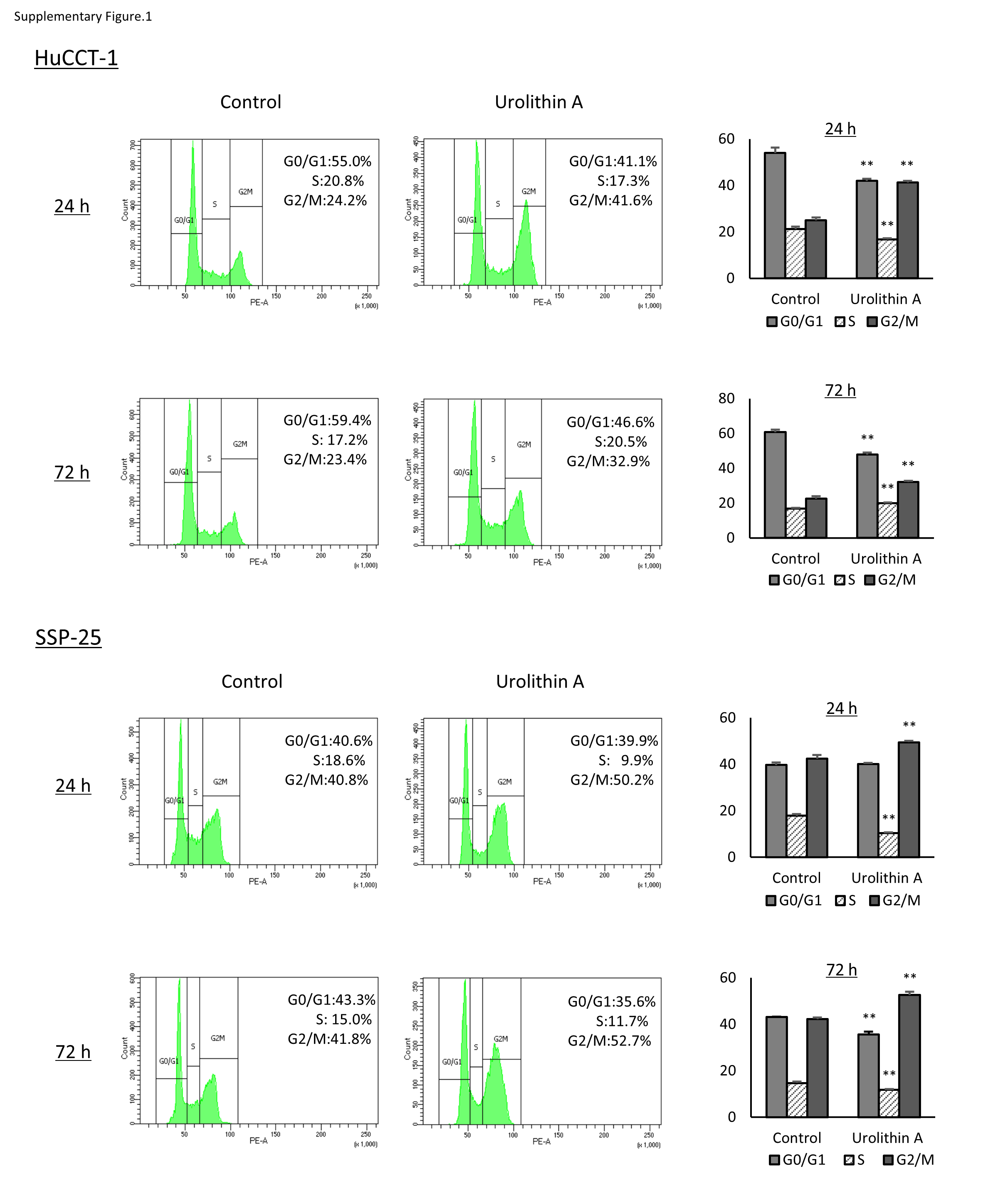

Supplement: Supplementary Figure 1 — UA treatment inhibits cell proliferation and induces G2/M phase cell cycle arrest in cholangiocarcinoma cell lines. HuCCT-1 and SSP-25 cells were treated with 0 or 40 μmol/L UA for 24 and 72 h. Cell cycles were determined using flow cytometry. Data represent the means of three independent experiments. Bars, standard deviation; **P < 0.01. [file Image_1.tif]

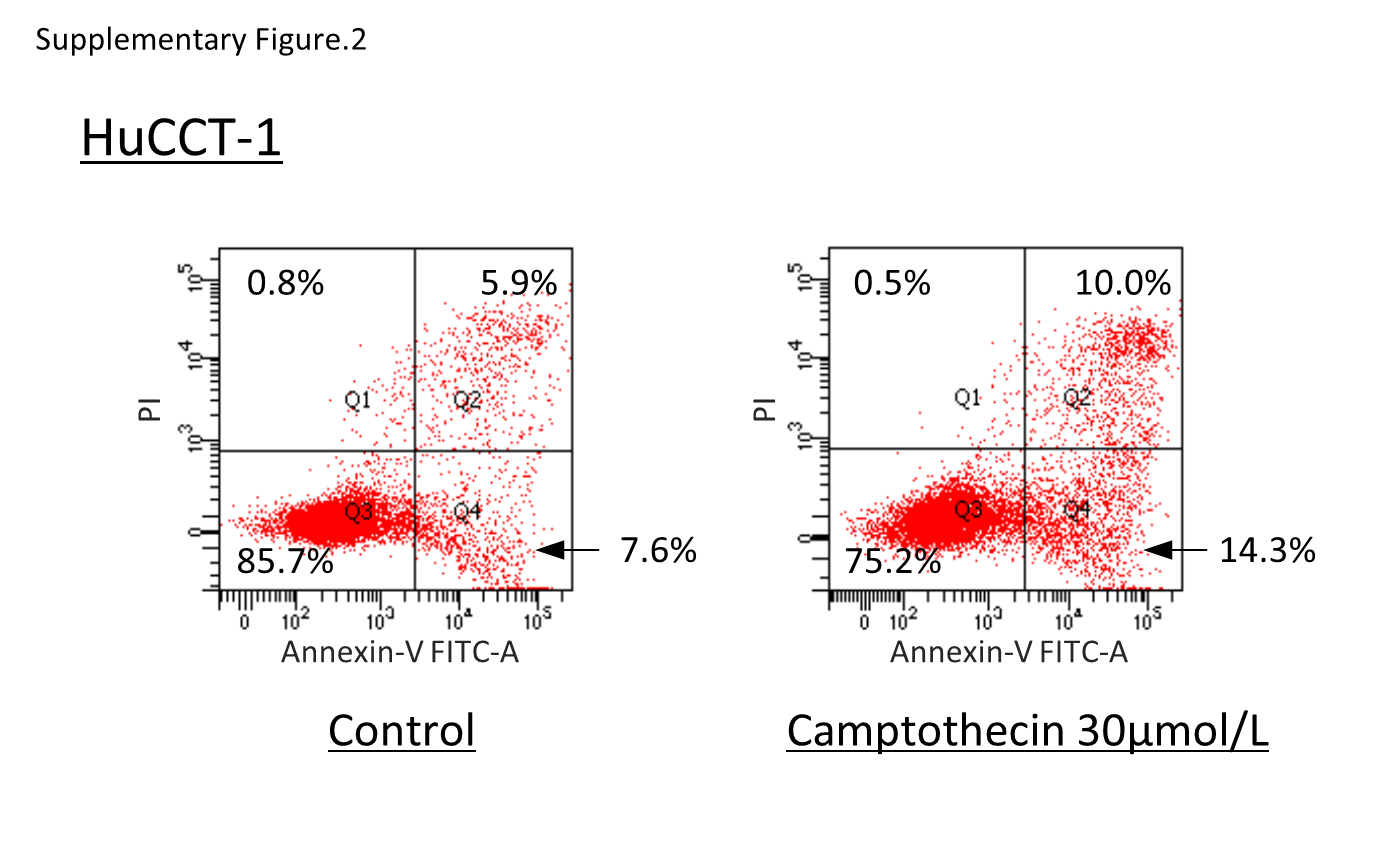

Supplement: Supplementary Figure 2 — Positive control for the apoptosis assay. HuCCT-1 cells were treated with 30 μmol/L Camptothecin for 24 h, and then stained with annexin-V FITC and PI. Apoptosis cells were evaluated using flow cytometry. [file Image_2.tif]

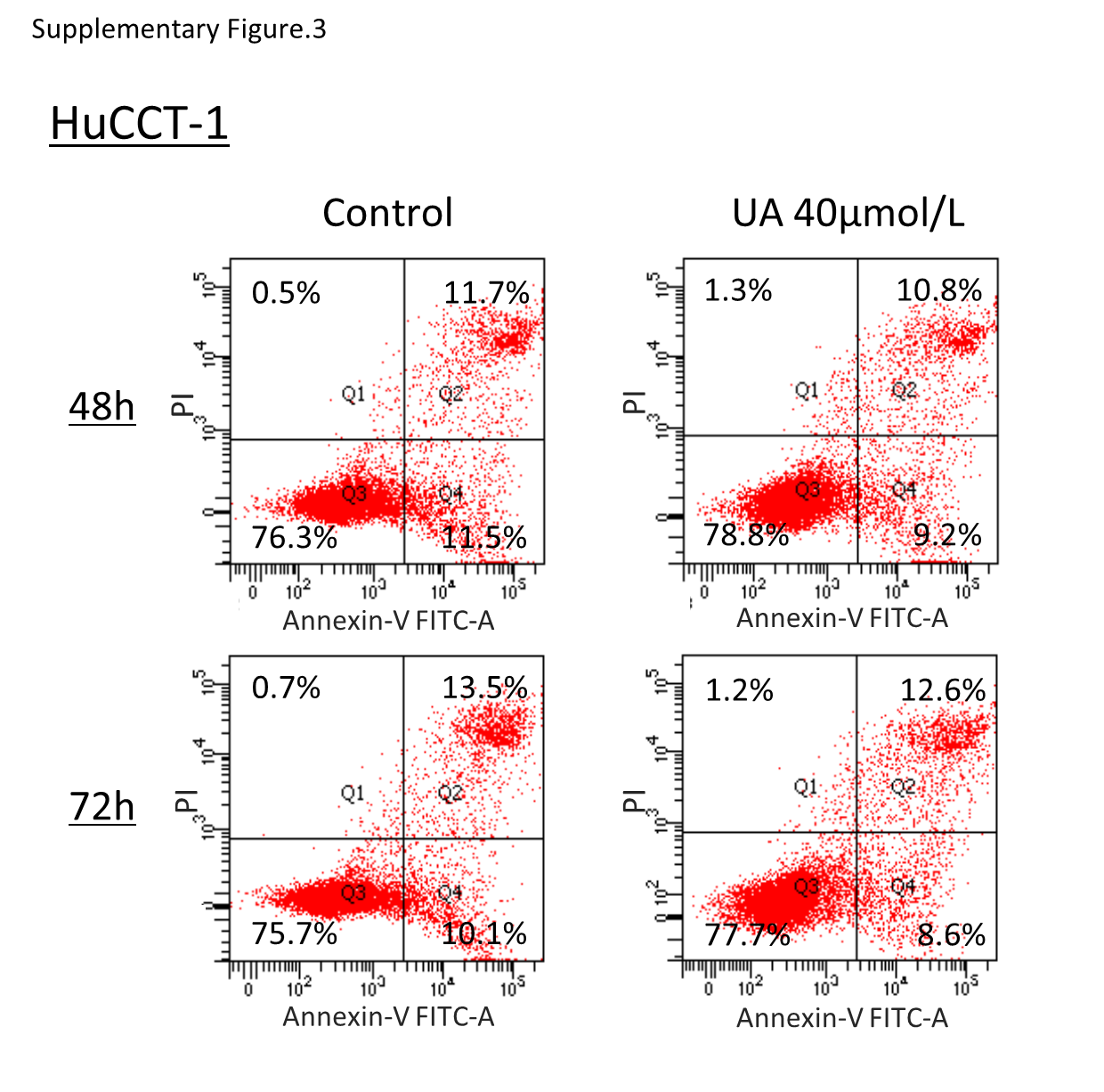

Supplement: Supplementary Figure 3 — Effects of UA on apoptosis progression. HuCCT-1 cells were treated with 0 or 40 μmol/L UA for 48 or 72 h, and then stained with annexin-V FITC and PI. Apoptosis cells were evaluated using flow cytometry. [file Image_3.tif]
